# The presentation of Legg−Calvé−Perthes disease in females

**DOI:** 10.1007/s11832-015-0671-y

**Published:** 2015-07-26

**Authors:** Andrew G. Georgiadis, Mark A. Seeley, Joseph L. Yellin, Wudbhav N. Sankar

**Affiliations:** Division of Orthopedic Surgery, The Children’s Hospital of Philadelphia, 34th and Civic Center Blvd, Philadelphia, PA 19104 USA

**Keywords:** Legg−Calvé−Perthes, Perthes disease, Osteonecrosis femoral head, Late presentation

## Abstract

**Purpose:**

Legg−Calvé−Perthes disease (LCPD) is uncommon in girls. The presentation of LCPD in female patients has been reported as later in onset and associated with certain high-impact activities. Our aim is to characterize the presentation of female LCPD at a large center, with particular attention to the clinical and radiographic features of late-onset disease (>ten years of age). We perceived an increasing burden of late-onset disease with adult-like radiographic features.

**Methods:**

All patients presenting to a single large urban children’s hospital from 1990−2014 with a diagnosis of LCPD were reviewed. Demographic, clinical, and radiographic data for all female patients were examined and compared to historical norms.

**Results:**

Four-hundred and fifty-one patients presented with LCPD in the study period, of which 82 (18.2 %) were female. The average age at presentation was 6.58 years in girls, which is similar to the classically reported mean age. Fourteen patients participated in high-impact repetitive activities or those with deep flexion and abduction, although few were late presenters. There were four female patients who presented for initial diagnosis >ten years of age.

**Conclusions:**

There was a paucity of late-onset LCPD in girls in the study population, and the females with LCPD had a very similar age and character to their presentation as did males. Although their presentation is infrequent, three of four older females with LCPD were engaged in high-level physical activity, and their disease may be attributed to high-impact, repetitive athletics.

**Level of evidence:**

Case series, Level IV.

## Introduction

Legg−Calvé−Perthes disease (LCPD) is an avascular necrosis of the developing femoral head, with an average age of onset between 4 and 9 years. Affected patients are disproportionately male by a 4:1 ratio [1]. The etiology of LCPD is unknown but is thought to result from vascular congestion and/or coagulation abnormalities in the circulation of the proximal femur [2, 3]. The diagnosis has been associated with behavioral disorders [4], cigarette smoking [5], femoral and acetabular morphology [6], genitourinary anomalies [7], genetics [8], geography [1], childhood deprivation [9, 10], and high-impact activities such as gymnastics [11, 12].

Although there are numerous reports on the presentation of LCPD in males, there are only a few studies that explicitly examine its presentation in females. Some of these studies have reported that female patients can present differently than male patients [11, 13–15]. Since girls have earlier proximal femoral physeal closure and less remodeling potential, the consequences of late presentation or high-risk activities may be clinically important. Gymnasts and dancers have been specifically reported to have disproportionate late-onset presentations and generally poor outcomes, with repetitive microtrauma theorized as the underlying cause of osteonecrosis [11]. The sparse literature on the presentation of female patients with LCPD highlights the importance of further study.

We undertook a 24-year review at a single institution to examine the presentation of female patients with LCPD. We hypothesized that our female patients would present at an older age and later in the disease course relative to historically accepted norms. After our report observing several high-level gymnasts and dancers who presented with atypical femoral head avascular necrosis (AVN) in early adolescence [12], we wished to identify all female patients with late-onset LCPD and adult-style femoral head collapse over a much longer period. We sought to identify, characterize, and determine the prevalence of this clinical entity.

## Methods

**Fig. 1:**
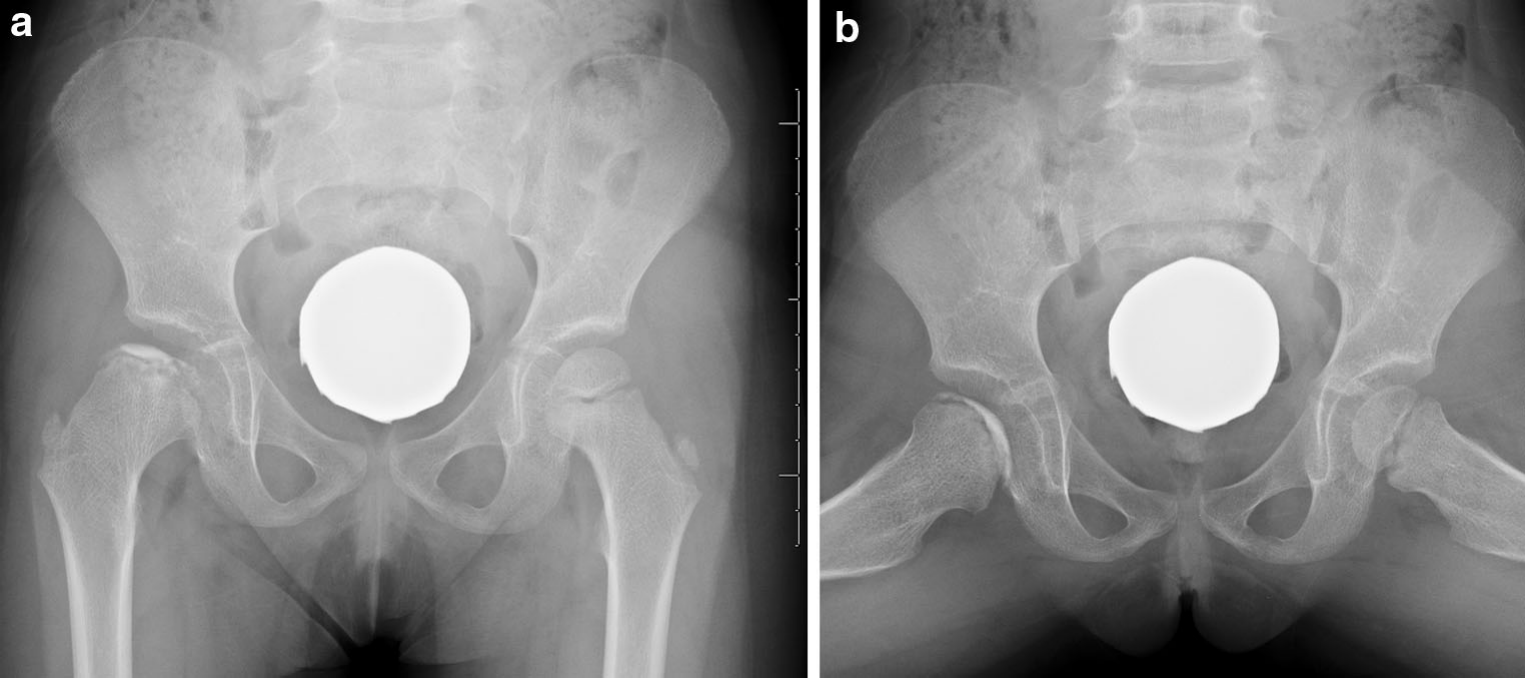
AP and frog lateral radiographs prototypical of the presentation of female Perthes disease, with femoral head fragmentation and loss of height of the lateral pillar

Institutional Review Board approval was obtained for this investigation at a single tertiary referral children’s hospital. International statistical classification of diseases revision 9 (ICD-9) codes 732.1 (‘juvenile osteochondrosis of the hip and pelvis’) and 733.42 (‘avascular necrosis of hip’) were queried for the years 1990–2014. The ICD-9 codes for AVN were utilized to ensure the collection of patients who may have been miscoded and exclusively identify those with truly idiopathic AVN. All inpatient, outpatient, and radiographic records were reviewed. All patients presenting with a diagnosis of LCPD were identified. Inclusion criteria were a diagnosis of Perthes disease, female gender, age <18 years, and a minimum of two serial anteroposterior (AP) and frog lateral pelvis radiographs to establish radiographic disease progression. We excluded patients who presented with a healed late-onset deformity (whose acute/initial presentation data was therefore unavailable), anyone with a history of hip pathology prior to presentation, and any patient with secondary AVN (e.g., sickle-cell disease, steroid use, chemotherapy). Data collected included demographics (race, body mass index [BMI], age of menarche, past medical history), presentation data (sidedness, age at presentation, chief complaint, presenting symptoms, presence/absence of limp, duration of symptoms, sports and activity history) and radiographic data (Fig. 1).

Clinical records were reviewed and radiographic staging performed (lateral pillar [16, 17] and Waldenström [18]) in a blinded fashion by two authors according to the methods of Guille et al. [14]. All pelvic radiographs were de-identified and blindly evaluated by the senior author to characterize AVN of the proximal femur and identify those with atypical patterns of collapse more akin to adult AVN (lack of lateral pillar involvement with more crescentic patterns of subchondral necrosis).

## Results

Over the study period, we identified 451 patients with LCPD treated at the study institution, of which 82 were female (18.2 %). The average age at presentation for girls was 6.58 years (range 1.91–10.4 years) and 6.61 years for boys. Sixty hips in fifty-eight female patients had the requisite clinical and radiographic records available, including sufficient data on clinical presentation and at least two serial AP pelvis radiographs, and these were included in the final review. All patients with incomplete records were treated between the years of 1990 and 1995.

Thirty-four involved hips were right (56.6 %) and 26 were left (43.4 %). The majority of patients presented with a documented limp (44/58; 75.8 %), with an average symptom duration of 4.3 months before referral to an orthopedic surgeon. The most commonly reported activity reported in clinical records was ‘school gym’ (six patients), with multiple other activities mentioned. Fourteen patients were participants in repetitive high-impact activities—gymnastics (5), dance (3), soccer (2), martial arts (2), ballet (1), cheerleading (1), skateboarding (1), and figure skating (1). The average BMI at presentation for all patients was 17.9 kg/m^2^ (Table 1).

**Table 1:** Activities as reported in the medical record of all girls presenting with LCPD

Activity	Number of patients^a^
‘School gym’	6
Gymnastics	5
Dance	3
Soccer	2
Martial arts	2
Cheerleading	1
Ballet	1
Skateboarding	1
Figure skating	1

^a^Four patients participated in two above activities and were calculated in both activities

Fragmentation stage radiographs were available for all hips. Female patients progressed through the radiographic stages of Waldenström (initial, fragmentation, reossification, healed) at mean ages of 5.9, 7.1, 7.5, and 10.1 years. At mid-fragmentation, most hips were classified as lateral pillar stage B (31/58; 53.4 %), followed by B/C (14/58; 24.1 %), A (8/58; 13.8 %), and C (4/58; 6.9 %). No patient experienced bilateral disease.

Four patients presented with idiopathic osteonecrosis between the ages of 10 and 16 years and three were involved in repetitive high-impact activities. One such patient had crescentic subchondral collapse in an adult pattern. Otherwise, no other females were found to have an adult pattern of femoral head collapse, and there were a small number of late presenters overall (4/58; 6.9 %).

## Discussion

Catterall reported that females have disproportionately greater head involvement in Perthes disease (Catterall III and IV), which could be responsible for ‘worse outcomes in females’, although boys and girls presenting within each stage would be bound to have a similar outcome regardless of sex [13]. The largest single-study review of female patients (155 hips in 136 patients) reported worse outcomes in females, suspected to be due to higher Catterall grade (i.e., femoral head involvement) [15]. Guille et al. performed a large review (122 hips in 105 females) in a similar geographic region to the present study, in which 18 % of Perthes disease patients were also female [14]. Girls with LCPD proceeded through the Waldenström radiographic staging at the same ages as male patients, albeit with earlier proximal femoral physeal closure. These patients had less opportunity for remodeling, and this was postulated as the etiology for inferior Stulberg [19] outcomes, although the study was not sufficiently powered to make this conclusion.

The average age of presentation in the current study was consistent with the largest epidemiologic review of available literature by Loder and Skopelja, where 18 % of patients were female (our study 18.2 %) and the overall average age of presentation was 6.58 years for girls and 6.61 for boys [1]. Similarly, most girls were lateral pillar B during fragmentation, and progressed through Waldenström staging at ages very similar to historical reports of boys [14, 16].

The underlying pathology in LCPD is unknown but considered multifactorial. Current theories have focused on the pathophysiology of vascular embarrassment to the proximal femur. Abnormalities (or variations) in vascular caliber and flow, likely secondary to endothelial dysfunction, have been independently related to rates of Perthes disease compared to controls [2]. The consistent reportage of a 4–5:1 male-to-female ratio among affected patients may suggest an endocrine, anatomic, or behavioral contribution accounting for differences in incidence between the sexes.

Anatomic variation plays an incompletely understood role in the development of Perthes disease. The intra-articular arterial ring of the proximal femur is more discontinuous in male patients, which may account for increased susceptibility to LCPD in males [20]. Mechanical compression of the lateral ascending cervical arteries may contribute to the disorder (i.e., in hyperactive patients or activities with repeated abduction), as this region of the trochanteric-neck junction is smaller in children, specifically before the age of eight years. Larson et al. described an ischial spine sign that could connote impingement on the vessels at the base of the femoral neck, but chronology of this deformity in the development of LCPD in their series could not be determined [6]. Furthermore, there is richer vascularity in the anterior femoral neck in black patients than in more susceptible Caucasians [20], which could explain the relative paucity of reported cases in blacks. Our proportion of non-Caucasian patients was small (six black, four Hispanic patients) despite a large urban hospital with significant minority populations.

Recently, high-level gymnastics has been implicated as a possible mechanical etiology of Perthes disease with repetitive microtrauma theorized as the potential pathomechanism of disease [11, 12]. One of the motivations for this study was to refute or deny this contention by determining an actual prevalence of this phenomenon. A number of patients in our series did have a documented history of activities at risk for repetitive microtrauma (e.g., gymnastics, ballet, dance), consistent with the growing numbers of females involved in high-level athletics in the United States. However, there was a surprising absence of females presenting with late-onset LCPD (>10 years of age), with only four such patients in our series over a 24-year period, and only three with such an activity history. The absence of this late-onset phenomenon in our study population may be explicable by the urban demographics of the study area which was comprised of more African–American residents, generally participating in gymnastic activities at lower rates [21, 22].

Another study objective was the characterization of late-onset patterns of disease in LCPD in females, which we suspected would not be well characterized by current Perthes disease classification systems and be more typical of adult forms of osteonecrosis. Joseph et al. described three patterns of LCPD in adolescents (both males and females)—late-onset, segmental collapse, and destructive, with poor radiological outcomes in all, postulating that these were a different and more severe entity. The distinction between LCPD and young-adult AVN is somewhat arbitrary if both are truly idiopathic, and we used a study design intending to capture and define all such radiographic forms of the disease. However, by the conventional criteria of ‘late-presenters’ we found only four patients aged >ten years of age who presented to our tertiary referral center, suggesting an uncommon phenomenon [12]. All other females presenting >ten years of age with the described ICD-9 codes had a history of corticosteroid use, chemotherapy, sickle-cell disease, or other established risk factor for AVN of the proximal femoral epiphysis and were therefore excluded. This finding was consistent with our surrounding urban demographics. The absence of a more widespread phenomenon when a large review was organized around its description was instructive vis-à-vis the cognitive biases operant in our perceptions of practice, our volume of patients, and the harsh reality of the medical records.

By design, this was not an outcomes study for a number of reasons. Long-term outcomes for LCPD have best been obtained in geographic areas with captive patient populations with exceptional long-term follow-up [23]. However, the most commonly reported outcomes instrument (Stulberg classification system [19]) has widely reported ranges of observer reliability and has been repeatedly questioned [24–26]. Furthermore, a 56-year retrospective review of LCPD in a similar geographic area to this study population was not sufficiently powered to detect differences in Stulberg outcome between the sexes, despite reviewing 575 cases at a single institution [14]. Moreover, our study reported the experience of a large tertiary care children’s hospital over a long period with many caregivers, without consistency in approach or treatment methodology from which to draw meaningful conclusions about outcome in this relatively small group. Since average ages of presentation and radiographic progression are well established for boys, we did not aim to recapitulate old data. Instead, our objective was to describe the presentation of Perthes disease in female patients.

Our intent was to characterize a new and ongoing phenomenon (clinically and radiographically) in the less-common female LCPD patient. We suspected that there was a demographically separate group of female patients who presented later than average with atypical patterns of femoral head fragmentation/collapse. The explosion of interest in female participation in particular sports was one theory for the anticipated demographic shift. Although we found a low burden of this phenomenon, we did identify a small group of highly active females who presented with late-onset disease. In our study area, LCPD presents in girls at a typical age in a typical radiographic pattern, while late presentation may be linked to trauma from repetitive, high-level athletics.
